# White meat consumption and risk of cardiovascular disease and type 2 diabetes: a systematic review and meta-analysis

**DOI:** 10.29219/fnr.v67.9543

**Published:** 2023-12-28

**Authors:** Alfons Ramel, Bright I. Nwaru, Christel Lamberg-Allardt, Birna Thorisdottir, Linnea Bärebring, Fredrik Söderlund, Erik Kristoffer Arnesen, Jutta Dierkes, Agneta Åkesson

**Affiliations:** 1 Faculty of Food Science and Nutrition, University of Iceland, Reykjavik, Iceland; 2 Krefting Research Centre, Institute of Medicine, University of Gothenburg, Gothenburg, Sweden; 3 Department of Food and Nutrition, University of Helsinki, Helsinki, Finland; 4 Health Science Institute, University of Iceland, Reykjavik, Iceland; 5 Department of Internal Medicine and Clinical Nutrition, Institute of Medicine, Sahlgrenska Academy, University of Gothenburg, Gothenburg, Sweden; 6 Unit of Cardiovascular and Nutritional Epidemiology, Institute of Environmental Medicine, Karolinska Institutet, Solna, Sweden; 7 Department of Nutrition, Institute of Basic Medical Sciences, University of Oslo, Oslo, Norway; 8 Centre for Nutrition, Department of Clinical Medicine, University of Bergen, Bergen, Norway; 9 Department of Laboratory Medicine and Pathology, Haukeland University Hospital, Bergen, Norway

**Keywords:** white meat, meta analysis, systema’c review, cardiovascular disease, type 2 diabetes, Nordic Nutri*on Recommenda*ons

## Abstract

**Objectives:**

The aim was to systematically review the associations among white meat consumption, cardiovascular diseases (CVD), and type 2 diabetes (T2D).

**Methods:**

Databases MEDLINE, Embase, and Cochrane Central Register of Controlled Trials and Scopus were searched (15th October 2021) for randomized intervention trials (RCTs, ≥ 4 weeks of duration) and prospective cohort studies (≥12 month of follow-up) assessing the consumption of white meat as the intervention/exposure. Eligible outcomes for RCTs were cardiometabolic risk factors and for cohorts, fatal and non-fatal CVD and incident T2D. Risk of bias was estimated using the Cochrane’s *RoB2* and *Risk of Bias for Nutrition Observational Studies*. Meta-analysis was conducted in case of ≥3 relevant intervention studies or ≥5 cohort studies using random-effects models. The strength of evidence was evaluated using the World Cancer Research Fund’s criteria.

**Results:**

The literature search yielded 5,795 scientific articles, and after screening 43 full-text articles, 23 cohort studies and three intervention studies were included. All included intervention studies matched fat content of intervention and control diets, and none of them showed any significant effects on the selected outcomes of white meat when compared to red meat. Findings from the cohort studies generally did not support any associations between white meat intake and outcomes. Meta-analyses were conducted for CVD mortality (RR: 0.95, 95% CI: 0.87–1.02, *P* = 0.23, I^2^ = 25%) and T2D incidence (RR: 0.98, 95% CI: 0.87–1.11, *P* = 0.81, *I*^2^ = 82%).

**Conclusion:**

The currently available evidence does not indicate a role, beneficial or detrimental, of white meat consumption for CVD and T2D. Future studies investigating potentially different health effects of processed versus unprocessed white meat and substitution of red meat with white meat are warranted.

**Registration:** Prospero registration CRD42022295915.

## Popular scientific summary

- Red meat can be a good source of essential nutrients, but excessive consumption of red meat can lead to undesirable high intakes of saturated fatty acids, iron as well as nitrate and has been linked to cardiovascular disease and type 2 diabetes.
- White meat (e.g., chicken, turkey) is one alternative to red meat and it usually contains less fat and iron. However, little is known about health effects of white meat.
- In this systematic review, we summarized the available evidence on white meat, cardiovascular disease and type 2 diabetes. All together, 26 studies were included in this analysis. Taken together, the results indicate that consumption of white meat does neither beneficially nor detrimentally affect the risk for these two diseases.

Meat consumption is common throughout the world, and meat production has tripled during the last 50 years (1). The average meat consumption is high in the Nordic countries (more than 100 g/day) (2–4), and most of the intake derives from red meat. Meat can be a good source of essential nutrients, for example, iron and vitamin B_12_ (5), but excessive consumption of meat and meat products can lead to undesirable high intakes of saturated fatty acids (6), iron (7), and nitrate (8). Consequently, high intake of red meat and meat products is a risk factor for several types of cancer (9), type 2 diabetes (T2D) (10), and cardiovascular disease (11, 12). Red meat consumption, in particular from beef, has also been criticized for its high ecological footprint, which is a measure of resources required to produce a given good and the wastes generated (13).

White meat (e.g. chicken and turkey) is one alternative to red meat. It usually contains less fat and iron (14) and exerts a lesser effect on the emission of greenhouse gases (11). However, less is known about the health effects of white meat. Available systematic reviews based on cohort studies suggest that white meat may protect against all-cause mortality (15), stroke (16), and cancer (17), although the evidence is unclear for heart disease and T2D (15).

As part of the process of updating national dietary reference values and food-based dietary guidelines, the Nordic Nutrition Recommendations 2022 project (NNR 2022) selected several topics for systematic reviews, one of these being the health effects of white meat (18). The aim of the present study was to systematically summarize the available evidence on white meat, cardiovascular disease (CVD), and T2D.

## Methods

The systematic review procedure followed an *a priori* determined systematic review protocol made for the NNR 2022 (19, 20), which is in agreement with the Preferred Reporting Items for Systematic Reviews and Meta-analyses (PRISMA) (21, 22). The NNR 2022 Committee established detailed research questions, including characterization of the study population, intervention/exposure, control, outcome, timeframe, study design, and settings (PI/ECOTSS) (Supplementary Table 1). The employed methods were registered in a PROSPERO protocol (CRD42022295915). The Nordic Council of Ministers and governmental food and health authorities of Norway, Finland, Sweden, Denmark, and Iceland funded this study (23).

### Eligibility criteria

We included prospective cohort studies and randomized controlled trials (RCTs) that included adults older than 18 years as the study population. The intervention/exposure was the consumption of white meat (i.e. poultry, chicken, turkey, duck, and goose, but not fish), whereas the comparator was red meat in intervention studies and no or low consumption of white meat in cohort studies. The minimum length of RCTs was 4 weeks, and the minimum follow-up length in the cohort studies was 1 year. We considered the following outcome variables for RCTs: insulin resistance, HBA1c, fasting glucose and insulin, blood pressure, total cholesterol, low density lipoprotein, high density lipoprotein, and triglycerides.

For cohort studies, the following outcomes were considered: major incident fatal and non-fatal CVD (combined or separate: myocardial infarction, stroke, coronary heart disease, and coronary artery bypass graft), CVD mortality, and incident T2D.

### Information sources and search strategy

An extensive search using MEDLINE, EMBASE, Cochrane Central of Controlled Trials, and Scopus was conducted by a senior librarian from the University of Oslo, Library of Medicine and Science, on 15th of October 2021. The search strategy (Supplementary Table 2) was not limited by publication date or language and was established together with the authors. The reference sections of the included studies were also examined to find potentially new eligible studies.

### Selection and data collection process

Papers found in the search were transferred to Endnote for the removal of duplicate publications. After de-duplication, the records were exported to Rayyan, where two reviewers (AR and EKA) independently screened the title and/or abstracts of the records. An article was included into full-text screening when at least one of the two authors voted for the inclusion of the paper. As the next step of the process, the librarians retrieved the full text of the papers, which were then examined by the two reviewers. Disagreements during the literature screening were resolved by discussion and by the support from the senior author (AÅ).

Key data from the publications were extracted into a data extraction form developed for this project by three independently working reviewers (FS, LB, and BN). Any disagreement between reviewers was resolved by discussion. The following variables were extracted from the included publications: full reference, participants and settings, interventions/exposures, outcomes, main results, confounding variables, dietary intake levels/dose, food source, method for dietary assessment, validation of dietary assessment method, food composition database used, and assessment of nutrition status.

### Risk of bias assessment

Assessment of risk of bias was done independently by two reviewers (JD and AR) using the Cochrane’s *Risk of bias 2.0* (24) for intervention trials and USDA’s *Risk of Bias for Nutrition Observational Studies* (RoB-NObS) (25) for prospective observational studies. Risk of bias was categorized as low, some concerns, or high for intervention studies, and low, moderate, serious, and critical for observational studies. Risk of bias for each study is displayed in a graphical way using the web app *Risk-of-bias VISualization* (26).

### Synthesis methods

In accordance with the guidelines for systematic reviews, meta-analyses were considered if deemed appropriate to combine/pool the different studies, but only when more than three independent RCTs or five cohort studies exist (27–29). When a sufficient number of studies were available, a random-effects meta-analysis with the generic inverse variance method was conducted using *Review Manager* (RevMan; The Cochrane Collaboration, 2020), version 5.4.1. When a study reported odds ratio, it was converted to relative risk using the online conversion tool ClinCalc (30) based on an article from Zhang et al. (31). Potential heterogeneity between studies was quantified using the *I*^2^ statistic, which estimates (range 0–100%) the proportion of variance in the pooled estimates attributable to differences in estimates between studies included in the meta-analyses. Pooled risks are shown using forest plots. Due to the low number of included publications, risk of publication bias using funnel plots could not be assessed.

### Certainty assessment

We used the World Cancer Research Fund’s grading (convincing, probable, limited – suggestive, limited – no conclusion, substantial effects unlikely) in order to categorize the strength of the available evidence (19, 23) based on study quality (risk of bias), quantity, consistency, and precision (details for grading can be seen in Supplementary Table 3).

## Results

### Study selection search results

**Fig. 1:**
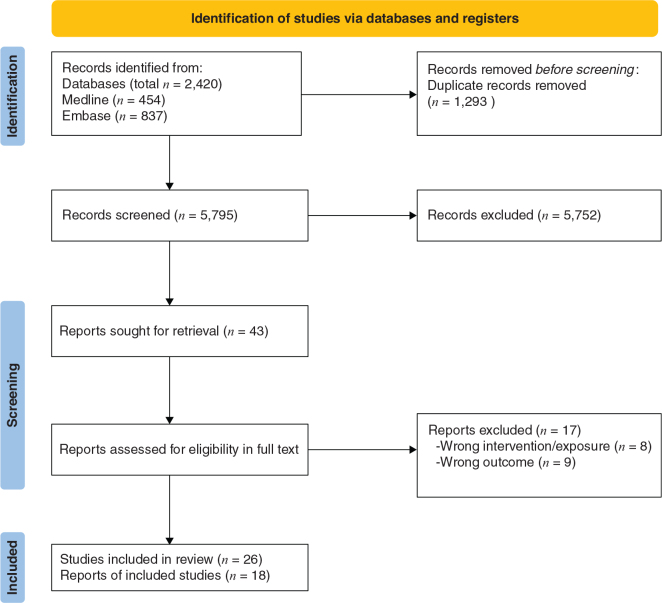
PRISMA flow chart of the article selection process.

As outlined in Fig. 1, a total of 5,795 records were retrieved from the database searches after de-duplication; of which 5,752 were excluded after title and/or abstract screening. Of the 43 full-text papers evaluated, three intervention studies (32–34) and 23 prospective cohort studies (35–57) met the criteria to be included in the review.

### Study characteristics

**Fig. 2:**
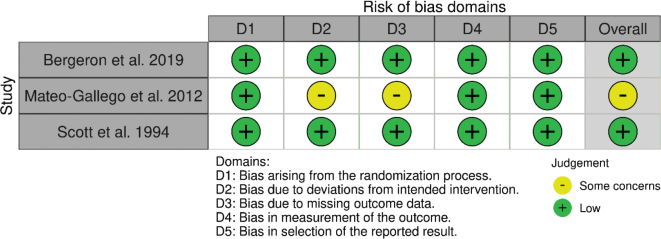
RoB of included intervention studies.

The three included intervention studies (32–34) investigated 36–177 adults each with an intervention period of 4 to 5 weeks and measured cardiometabolic risk factors (for details see Table 1). The RoB was judged to be low for two, but there were some concerns for one of the studies (see Fig. 2).

**Table 1:** Selected characteristics of the included studies.

Author and year	Country	Design	Treatment/exposures	Dietary assessment methods	Participants	Age at inclusion/start of intervention	Intervention period/follow-up time	Type of outcome	Confounders adjusted for
**Intervention studies**									
Bergeron et al. 2019	USA	Two crossover-RCTs, one low SFA, and one high SFA	White meat: ∼12 E% Red meat: ∼12 E% Non-meat: ∼15 E% Low-SFA: ∼7 E% High-SFA: ∼14 E%	N/A (given all food, compliance assessed through N and urea in 24 h urine)	177	High-SFA: 45 ± 12 years Low-SFA: 42 ± 13 years	1 month/diet	Blood lipids, blood pressure, and glucose metabolism	n.a.
Mateo-Gallego et al. 2012	Spain	Crossover-RCT	Instructed to consume 125 g of meat, 3 day/week, for 5 weeks	3 days food record/diet period	36	≥18 y (median 71 years, interquartile range 33–79)	5 weeks/diet	Blood lipids and blood pressure	n.a.
Scott et al. 1994	USA	Parallel RCT	226.8 g of cooked chicken	1	80	20–55 years	5 weeks	Blood lipids	n.a.
**Cohort studies – CVD**									
Bernstein et al. 2010	USA	Pros. cohort	Median: Q1: 0.07 serving/day Q2: 0.14 serving/day Q3: 0.24 serving/day Q4: 0.40 serving/day Q5: 0.56 serving/day	FFQ	121,700	30–55	26 years (2,050,071 person-years)	Fatal CHD and non-fatal myocardial infarction	Age, time period (13 periods), total energy, cereal fiber, alcohol, trans fat, BMI, cigarette smoking, menopausal status, parental history of early myocardial infarction, multivitamin use (fifths of years), vitamin E supplement use (yes/no), aspirin use, and physical exercise
Bernstein et al. 2012	USA	Pros. cohort	Median (only Q1, Q3, and Q5 presented): HPFS: Q1: 0.14 serving/day Q3: 0.40 serving/day Q5: 0.72 serving/day NHS: Q1: 0.14 serving/day Q3: 0.28 serving/day Q5: 0.54 serving/day	FFQ	Pooled: 173,229 NHS: 121,700 HPFS: 51,529	NHS: 30–55 years HPFS: 40–75 years	NHS: 26 years (2,041,679 person-years) HPFS: 22 years (833,660 person-years)	Stroke	Stratified on age and time period and includes: BMI, cigarette smoking, physical exercise, parental history of early myocardial infarction, menopausal status in women, multivitamin use, vitamin E supplement use, aspirin use at least once per wk, total energy, cereal fiber, alcohol, transfat, fruit and vegetables, and other protein sources
Farvid et al. 2017	Iran	Pros. cohort	Median: Q1: 0.11 serving/day Q2: 0.33 serving/day Q3: 0.54 serving/day Q4: 0.78 serving/day Q5: 1.33 serving/day	FFQ (interview administered)	50,045	36–85	Median: 8.1 years (339,867 person-years), total 11 years	CVD, CHD, and stroke	Gender, age; ethnicity; education; marital status; residency; smoking; opium use; alcohol; BMI; systolic blood pressure; occupational physical activity; family history of cancer; wealth score; medication; and energy intake
Haring et al. 2014	USA	Pros. Cohort	Median: Q1: 0.1 servings/day Q2: 0.1 servings/day Q3: 0.3 servings/day Q4: 0.4 servings/day Q5: 0.8 servings/day	FFQ (interview administered)	15,792	45–64	Median 22 years	Myocardial infarction or death from CHD	Age, sex, race, study center, total energy intake, smoking, education, systolic blood pressure, use of antihypertensive medication, HDLc, total cholesterol, use of lipid lowering medication, body mass index, waist-to-hip ratio, alcohol intake, sports-related physical activity, leisure-related physical activity, carbohydrate intake, fiber intake, and magnesium intake
Haring et al. 2015	USA	Pros. cohort	Female range, g/day Q1: <56.0 Q2: 56.0–63.7 Q3: >63.7–70.8 Q4: 70.8–79.6 Q5: >79.6 Male range, g/day Q1: <62.4 Q2: 62.4–70.1 Q3: 70.2–77.2 Q4: 77.2–85.8 Q5: >85.78	FFQ (interview administered)	11,601	45–64	Median 22.7 years	Stroke incidence	Age, sex, race, study center, total energy intake, smoking, cigarette years, education, systolic blood pressure, use of antihypertensive medication, HDLc, total cholesterol, use of lipid lowering medication, body mass index, waist-to-hip ratio, alcohol intake, sports-related physical activity, leisure-related physical activity, carbohydrate intake, fiber intake, fat intake, and magnesium intake
Kappeler et al. 2013	USA	Pros. cohort	Servings/month	FFQ	33,944	18 or older	22 years	CVD mortality	Age, race, sex, cigarette smoking, alcohol consumption, physical activity, socioeconomic status, BMI, marital status, fruit and vegetables intake, history of hypertension, diabetes, hypercholesterolemia, use of aspirin and ibuprofen, use of mineral and vitamin supplements, family history of diabetes or hypercholesterolemia, hormone replacement therapy and oral contraceptive use (in women)
Key et al. 2019	France, Greece, Italy, The Netherlands, Spain, UK, Sweden, Denmark, and Norway	Pros. cohort	Median: Men: 16 g/day Women: 14 g/day	FFQ	518,502	Mean (SD): Men: 52.7 (10.3) Women: 51.3 (9.8)	Mean 12.6 years	IHD as composite of first non-fatal myocardial infarction or death from IHD	Age, smoking status, number of cigarettes per day, history of diabetes mellitus, previous hypertension, prior hyperlipidemia, Cambridge physical activity index, employment status, level of education completed, BMI, current alcohol consumption; and observed intakes of energy, fruit, and vegetables combined; sugars, fiber from cereals, and each other food; and stratified in the analysis by sex and EPIC center
Lee et al. 2013	Bangladesh, China, Japan, Korea, and Taiwan	Pooled (IPD?) pros. cohorts	Tertiles of intake in g/day	FFQ	NI (but probably 305,365)	Age ranged from 18 to 92 years in the different studies	Mean ranged from 6.6 to 15.6 years	NI	Age, BMI, education, smoking habit, rural/urban residence, alcohol intake, fruit and vegetable intakes, and total energy intake
Nagao et al. 2012	Japan	Pros. cohort	Median: Men: Q1: 1.9 g/day Q2: 3.3 g/day Q3: 10.2 g/day Q4: 13.3 g/day Q5: 27.3 g/day Women: Q1: 1.5 g/day Q2: 4.2 g/day Q3: 8.6 g/day Q4: 11.3 g/day Q5: 22.4 g/day	FFQ	110,792	40–79 years	Median 18.4 years (820,076 person-years)	Mortality from ischemic heart disease, stroke, and total cardiovascular disease	Age, BMI, smoking status, ethanol intake, perceived mental stress, walking time, sports participation time, education years, history of hypertension and diabetes, total energy, and energy-adjusted food (rice, fish, soy, vegetables, and fruits) intakes.
Park et al. 2017	South Korea	Pros. cohort	Q1: 0 servings/week Q2: 0.17 servings/week Q3: 0.35 servings/week Q4: 0.57 servings/week Q5: 1.41 servings/week	110-Item semi-quantitative FFQ	10,030	40–69 years	Median 7.8 years	CVD events	Age, sex, total energy intake, BMI, alcohol use, smoking, physical activity, education status, household income, residential area, and fruit and vegetable intakes
Rohrmann et al. 2013	France, Italy, Spain, The Netherlands, United Kingdom, Greece, Germany, Sweden, Norway, and Denmark	Pros. cohort	Median: Men: 15.1 g/day Women: 12.6 g/day	FFQ, seven-day food record (UK) and quantitative questionnaire combined with a seven-day menu book (Sweden)	511,781	Median: Men: 52.3 Women: 50.9	Median 12.7 years with a maximum of 17.8 years; median follow-up time was 8.5 years in cases and 12.9 years in non-cases	CVD mortality	Stratified by age, sex, study center, adjusted for education, body weight, body height, total energy intake, alcohol consumption, physical activity, smoking status, and smoking duration
Sauvaget et al. 2003	Japan	Pros. cohort	Never ≤once/week 2–4 times/week Almost daily	22-item FFQ	55,650	Mean: 56 years (range 34–103)	16 years	Mortality?	HR stratified by sex and birth cohort, and adjusted for city, radiation dose, self-reported body mass index, smoking status, alcohol habits, education level, history of diabetes, or hypertension
Takata et al. 2013	China	Pros. cohort	Women: Q1: 11.9 ± 0.15 Q5: 19.9 ± 0.15 Men: Q1: 11.9 ± 0.17 Q5: 22.3 ± 0.18	FFQ	136,424 (women: 74,941, men: 61,483)	Women: Q1: 55.2 ± 9.5 Q5: 50.5 ± 8.2 Men: Q1: 58.2 ± 10.3 Q5: 52.7 ± 8.7	Median: Women: 11.2 years (803,265 person-years) Men: 5.5 years (334,281 person-years)	Mortality from ischemic heart disease, hemorrhagic stroke, and ischemic stroke	Age at baseline, total caloric intake, income, occupation, education, comorbidity index, physical activity level, total vegetable intake, total fruit intake, fish intake, and red meat or poultry intake where appropriate, smoking history (ever/never smoking for women and pack-years of smoking for men), and alcohol consumption (for men only)
van den Brandt et al. 2019	Netherlands	Case-cohort	Median (g/day): 0: 0 <10: 4.3 <20: 13.2 20+: 22.8	Semi-quantitative FFQ	120,852 (men: 58,279, women: 62,573)	55–69 years	10 years	Mortality from CVD	Age at baseline, sex, cigarette smoking status, number of cigarettes smoked per day, years of smoking, history of physician-diagnosed hypertension and diabetes, body height, BMI, non-occupational physical activity, highest level of education, intake of alcohol, vegetables and fruit, nuts, energy, use of nutritional supplements, and, in women, postmenopausal hormone replacement therapy
**Cohort studies – T2D**									
Du et al. 2020	China	Pros. cohort	Servings per day. Consumption weekly, monthly, and never/rarely	FFQ (interview administered)	512,713	Mean (SD): 51.2 (10.5) years	9 years	T2D	Age-at-risk, sex, region, education, income, smoking, alcohol consumption, physical activity, family history of diabetes, fresh fruit consumption, red meat, fish, and BMI
InterAct. 2013	Denmark, France, Germany, Italy, the Netherlands, Spain, Sweden, and UK	Case-cohort	Mean (SEM): Q1: 8.7 (0.4) g/day Q2: 15.7 (0.4) g/day Q3: 20.6 (0.4) g/day Q4: 26.1 (0.4) g/day Q5: 37.7 (0.4) g/day	Country-specific questionnaires	340,234	20–80	Mean 11.7 years	T2D	Stratified by center. Adjusted for sex, energy intake, smoking status, alcohol consumption, physical activity, educational level, and BMI
Kurotani et al. 2013	Japan	Pros. cohort	Median: Men: Q1: 0.0 g Q2: 5.1 g Q3: 9.6 g Q4: 20.1 g Women: Q1: 0.0 g Q2: 4.5 g Q3: 8.6 g Q4: 17.8 g	FFQ	113,403	40–69	5 years	T2D	Age, public health center area, BMI, smoking status, alcohol consumption, total physical activity, the history of hypertension, coffee consumption, the family history of diabetes, Mg intake, Ca intake, rice intake, fish intake, vegetable intake, soft drink consumption, energy intake, and saturated fat
Männistö et al. 2010	Finland	Prospective cohort within an RCT study	Median: Q1: 2 g/day Q2: 8 g/day Q3: 10 g/day Q4: 14 g/day Q5: 14 g/day	FFQ	29,133	50–69	12 years	T2D	Age, intervention group, BMI, number of cigarettes smoked daily, smoking years, systolic blood pressure, diastolic blood pressure, serum total cholesterol, serum HDL-cholesterol, leisure-time physical activity, intakes of alcohol and energy, consumption of fruits, vegetables, rye, milk, and coffee
Montonen et al. 2005	Finland	Pros. cohort	Mean (SD): Non-cases: 2.6 (9.3) g/day Cases: 2.6 (13.1) g/day	Dietary history interview	4,304	Mean (SD) Non-cases: 51.7 (8.0) T2D cases: 53.7 (7.6)	23 years	T2D	Age, sex, body mass index, energy intake, smoking, family history of diabetes, and geographic area
Steinbrecher et al. 2011	USA	Pros. cohort	Median intake (g/4,184 kJ/day): Men: Fresh poultry: Q1: 5.98 Q2: 11.65 Q3: 16.83 Q4: 23.60 Q5: 38.18	FFQ	103,898	Median: 59	Mean 14 years, median 13.5 years	T2D	Ethnicity, education, BMI, physical activity, and total energy intake (log-transformed) as well as stratified by age at cohort entry
			Processed poultry: Q1: 0.00 Q2: 0.11 Q3: 0.53 Q4: 1.20 Q5: 2.85 Women: Fresh poultry: Q1: 6.46 Q2: 12.65 Q3: 18.37 Q4: 26.40 Q5: 43.24 Processed poultry: Q1: 0.00 Q2: 0.10 Q3: 0.42 Q4: 1.06 Q5: 2.42						
Talaei et al. 2017	Singapore	Pros. cohort	Mean (SD): Q1: 4.1 (6.2) Q4: 40.9 (15.5)	FFQ	54,341	Mean (SD): 55.2 (7.6)	Mean 10.9 years (494,741 person-years)		Age, sex, dialect, year of interview, educational level, body mass index, physical activity level, smoking status, alcohol use, baseline history of self-reported hypertension, adherence to the vegetable-, fruit-, and soy-rich dietary pattern, total energy intake, and heme iron intake
van Woudenbergh et al. 2012	Netherlands	Pros. cohort	Median: 0: 0 g/day >0–≤9.1: 6.3 g/day >9.1–≤18.0: 13.9 g/day >18.0: 27.6 g/day	170-Item FFQ	7,983	Mean (SD): 67.3 (8) years	Median 12.4 years	T2D	Age, sex, smoking, diet prescription, family history of diabetes, intake of energy, energy-adjusted carbohydrates, energy-adjusted polyunsaturated fatty acids, energy-adjusted fiber, energy-adjusted milk, energy-adjusted cheese, soya, fish, alcohol, tea, and intakes of red meat and processed meat
Villegas et al. 2006	China	Pros. cohort	NI	77-Item FFQ	75,221	Mean (SD): 51.7 (8.97)	4.6 years (326,581 person-years)	T2D	Adjusted for age, kcals/day, BMI, WHR, smoking, alcohol consumption, physical activity, vegetable intake, income level, education level, occupation status, and hypertension. For analyses on all participants, chronic disease was also adjusted

**Fig. 3:**
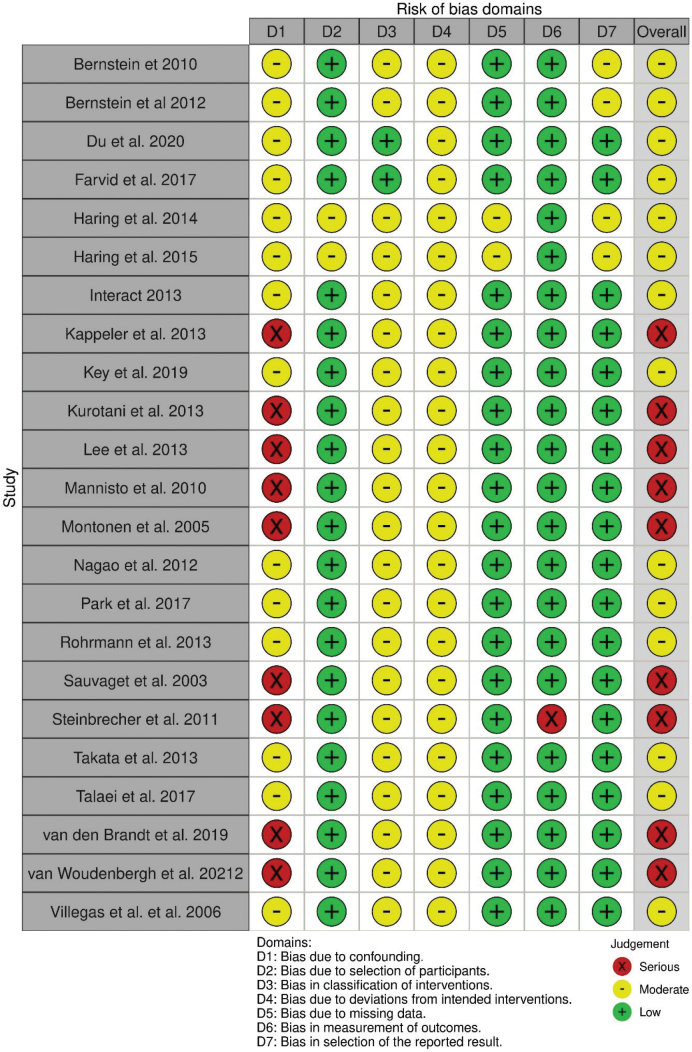
RoB of included cohort studies.

The 23 included observational studies (35–57) were all prospective cohort studies investigating from 4,304 up to 511,781 adults from Europe, Asia, and the USA with a follow-up ranging from 4.6 to 26 years (for details, see Table 1). The RoB was judged as moderate for 12 and serious for 11 of the included studies (see Fig. 3), mainly due to risk of bias related to confounding, selection of participants, and exposure assessment.

### Intervention studies

The effects of white meat compared to red meat on blood lipids were investigated by Bergeron et al. (32), Mateo-Gallego et al. (33), and Scott et al. (34). Effects on blood pressure were investigated by Bergeron et al. (32) and Mateo-Gallego et al. (33). Effects on glucose metabolism were investigated by Bergeron et al. (32) only. All of the included studies matched fat content of the intervention and control diets, and none of them showed any significant effects on the selected outcomes of white meat when compared to red meat (Table 2). Due to the low number of studies, no meta-analysis was conducted.

**Table 2:** Overview on included studies and individual study results.

Author and year	Intervention	Outcome measurements	Results	Interpretation	Overall risk of bias
**Intervention studies**					
Bergeron et al. 2019	White meat: ∼12 E% Red meat: ∼12 E%	TC, LDL, HDL, TG, SBP, DBP, and glucose	Red meat versus white meat (mmol/L): TC: -0.01 ± 0.04, *P* = 0.88; LDL: 0.001 ± 0.03, *P* = 0.98, HDL: -0.01 ± 0.01, *P* = 0.58; TG: -0.00 ± 0.02, *P* = 0.97; SPB (mmHg): 109 ± 12 versus 109 ± 12; DBP (mmHg): 69 ± 8 versus 69 ± 8; Fasting glucose (mmol/L): 5.36 ± 0.44 versus 5.37 ± 0.44; no significant differences	There was no difference in blood lipids between red meat and white meat consumption	Low
Mateo-Gallego et al. 2012	Instructed to consume 125 g of meat, 3 day/week, for 5 weeks	TC, LDL, HDL, TG, SBP, and DBP	Baseline versus lamb versus poultry: Total cholesterol (mg/L): 194 ± 61 versus 195 ± 71 versus 195 ± 61, *P* = 0.895; TAG (mg/L): 660 (420–900) versus 580 (430–915) versus 630 (495–1,030), *P* = 0.529; LDL-cholesterol: 116 ± 58 versus 119 ± 64 versus 119 ± 54, *P* = 0.463; HDL-cholesterol (mg/L): 554 ± 19 versus 526 ± 17 versus 518 ± 17, *P* = 0.009. HDL difference between lamb and poultry is not significant. No change in BP, data not shown	Poultry consumption did not affect blood lipids when compared to lamb but decreased HDL when compared to baseline diet	Some concerns
Scott et al. 1994	226.8 g of cooked chicken	TC, LDL, HDL, and TG	Changes in blood lipids after beef and chicken intervention compared to test diet: Total cholesterol (mmoL): 0.54 ± 0.40 versus 0.70 ± 0.52, both *P* < 0.002; TAG (mmoL): 0.02 ± 0.054 versus 0.08 ± 0.28, *P* = n.s.; LDL-cholesterol (mmoL): 0.46 ± 0.43 versus 0.55 ± 0.35, both *P* < 0.002; HDL-cholesterol (mmoL): 0.06 ± 0.11 versus 0.12 ± 0.15, *P* < 0.02; No significant differences between beef and chicken	Chicken consumption did not affect blood lipids when compared to beef but improved TC, LDL, and HDL when compared to baseline diet	Low

Author and year	Exposure	Results	Interpretation	Overall risk of bias
**CHD incidence**				
Bernstein et al. 2010	**Median intakes:** Q1: 0.07 serving/day Q2: 0.14 serving/day Q3: 0.24 serving/day Q4: 0.40 serving/day Q5: 0.56 serving/day	**Poultry and CHD incidence:** Q1: 1.00 (ref) Q2: 1.07 (0.96, 1.20), Q3: 0.91 (0.80, 1.04), Q4: 0.94 (0.83, 1.06), Q5: 0.92 (0.80, 1.06), RR 1 serving per day: 0.90 (0.75, 1.08)	Poultry intake was not associated with risk of CHD incidence	Moderate
Haring et al. 2014	**Median intakes:** Q1: 0.1 servings/day Q2: 0.1 servings/day Q3: 0.3 servings/day Q4: 0.4 servings/day Q5: 0.8 servings/day	**Poultry and CHD incidence:** Q1: 1 (ref), Q2: 0.83 (0.70, 0.99), Q3: 0.93 (0.75, 1.15), Q4: 0.88 (0.73, 1.06), Q5: 0.79 (0.64, 0.98), *P* for trend = 0.16	Higher poultry intake was associated with a lower risk of CHD incidence	Serious
Key et al. 2019	**Median intakes:** Men: 16 g/day Women: 14 g/day	**White meat and risk of ischemic heart disease:** Q1: 1.00 (ref); Q2: 1.00 (0.92–1.09), Q3: 0.99 (0.92–1.08), Q4: 1.00 (0.92–1.09), Q5: 1.01 (0.94–1.10), *P* = 0.77	Intake of white meat was not associated with risk of ischemic heart disease	Moderate
**CHD mortality**				
Farvid et al. 2017	**Median intakes:** Q1: 0.11 serving/day Q2: 0.33 serving/day Q3: 0.54 serving/day Q4: 0.78 serving/day Q5: 1.33 serving/day	**Poultry and CHD mortality** Q1: 1, Q2: 0.83 (0.66, 1.04,) Q3: 1.06 (0.85, 1.32), Q4: 0.92 (0.73, 1.15), Q5: 0.97 (0.77, 1.22), *P* = 0.90. **3 servings/week:** 1.00 (0.93, 1.08)	Intake of poultry was not associated with stroke, CVD, or CHD mortality	Moderate
Nagao et al. 2012	**Median intakes:** **Men:** Q1: 1.9 g/day Q2: 3.3 g/day Q3: 10.2 g/day Q4: 13.3 g/day Q5: 27.3 g/day **Women:** Q1: 1.5 g/day Q2: 4.2 g/day Q3: 8.6 g/day Q4: 11.3 g/day Q5: 22.4 g/day	**Poultry and mortality from ischemic heart disease: Men:** Q1: 1.00 (ref); Q2: 0.85 (0.58–1.25), Q3: 0.93 (0.63–1.37), Q4: 0.63 (0.41–0.96), Q5: 0.86 (0.60–1.23), *P* for trend= 0.405; **Women:** Q1: 1.00 (ref); Q2: 1.09 (0.72–1.66), Q3: 1.24 (0.78–1.98), Q4: 1.12 (0.72–1.74, Q5: 1.06 (0.69–1.62), *P* for trend= 0.888	Intake of poultry was not associated with risk of ischemic heart disease.	Moderate
**Stroke incidence**				
Bernstein et al. 2012	**Median intakes** (only Q1, Q3, and Q5 presented): HPFS: Q1: 0.14 serving/day Q3: 0.40 serving/day Q5: 0.72 serving/day NHS: Q1: 0.14 serving/day Q3: 0.28 serving/day Q5: 0.54 serving/day	**Stroke incidence and poultry intake:** **Men:** Q1: 1.00 (ref), Q2: 1.01 (0.85–1.20), Q3: 1.00 (0.84 –1.18), Q4: 1.06 (0.87–1.28), Q5: 0.97 (0.81–1.17), Per 1 serving per/day: 0.95 (0.70 –1.28) **Women:** Q1: 1.00 (ref), Q2: 1.01 (0.88–1.15), Q3: 0.91 (0.80–1.03), Q4: 0.91 (0.80–1.04), Q5: 0.82 (0.71–0.94), Per 1 serving/day: 0.61 (0.45–0.83)	Data suggest that stroke risk may be reduced by poultry intake, especially in women	Moderate
		**Men and women:** Q1: 1.00 (ref), Q2: 1.01 (0.91–1.12), Q3: 0.94 (0.85–1.04), Q4: 0.96 (0.86 –1.07), Q5: 0.87 (0.78–0.97), Per 1 serving per/day: 0.77 (0.62– 0.95)		
Haring et al. 2015	**Female range, g/day** Q1: <56.03 Q2: 56.03–63.65 Q3: >63.65–70.81 Q4: 70.82–79.57 Q5: >79.58 **Male range, g/day** Q1: <62.44 Q2: 62.44–70.14 Q3: 70.15–77.19 Q4: 77.20–85.77 Q5: >85.78	**Stroke incidence and poultry intake for both sexes:** Q1: 1 (ref), Q2: 0.90 (0.71, 1.15), Q3: 0.87 (0.65, 1.15), Q4: 0.90 (0.70, 1.16), Q5: 0.86 (0.65, 1.14), *P* for trend = 0.55	Poultry intake was not associated with stroke incidence	Serious
**Stroke mortality**				
Farvid et al. 2017	**Median intakes:** Q1: 0.11 serving/day Q2: 0.33 serving/day Q3: 0.54 serving/day Q4: 0.78 serving/day Q5: 1.33 serving/day	**Poultry and stroke mortality:** Q1: 1 (ref), Q2: 0.89 (0.68, 1.18), Q3: 0.98 (0.75, 1.30), Q4: 0.99 (0.75, 1.31), Q5: 1.06 (0.80, 1.39), *P* = 0.47 **3 servings/week:** 1.03 (0.94, 1.13)	Intake of poultry was not associated with stroke, CVD, or CHD mortality	Moderate
Sauvaget et al. 2003	**Intake categories:** Never ≤once/week 2–4 times/week Almost daily	**Poultry and stroke mortality, 4 categories:** Never: 1.00 (ref), ≤1 time/week: 0.88 (0.70, 1.10), 2–4 times/week: 0.99 (0.79, 1.25), Almost daily: 1.43 (0.98, 2.10), *P* for trend = 0.011	Intake of poultry was not associated with stroke mortality	Moderate
**CVD incidence**				
Park et al. 2017	**Median intakes:** Q1: 0 servings/week Q2: 0.17 servings/week Q3: 0.35 servings/week Q4: 0.57 servings/week Q5: 1.41 servings/week	**Poultry and CVD incidence:** Q1: 1.00 (ref), Q2: 0.98 (0.75–1.29), Q3: 0.89 (0.67–1.19), Q4: 0.99 (0.74–1.34), Q5: 0.68 (0.47–0.99), *P* for trend= 0.04	Intake of poultry was associated with lower risk of incident CVD	Moderate
**CVD mortality**				
Farvid et al. 2017	**Median intakes:** Q1: 0.11 serving/day Q2: 0.33 serving/day Q3: 0.54 serving/day Q4: 0.78 serving/day Q5: 1.33 serving/day	**Poultry and CVD mortality:** Q1: 1 (ref)m Q2: 0.93 (0.79, 1.10), Q3: 1.04 (0.89, 1.22), Q4: 0.95 (0.80, 1.12), Q5: 1.03 (0.87, 1.21), *P* = 0.63 **3 servings/week:** 1.01 (0.96, 1.07)	Intake of poultry was not associated with CVD mortality	Moderate
Kappeler et al. 2013	Servings/month	**White meat and CVD mortality:** 0/month: 1 (ref), 0–3/month: 0.95 (0.58–1.56), 4–8/month: 1.20 (0.77–1.88), 9–12/month: 1.01 (0.65–1.57), ≥13/month: 1.05 (0.65–1.71), P-trend 0.90	White meat intake was not associated with CVD mortality risk	Serious
Lee et al. 2013	**Tertiles of intake in g/day**	**Poultry and CVD mortality** **Men:** T1: 1.00 (ref), T2: 0.82 (0.66, 1.02), T3: 0.82 (0.64, 1.06), *P* for trend = 0.14 **Women:** T1: 1.00 (ref), T2: 0.97 (0.85, 1.09), T3: 1.05 (0.92, 1.18), *P* for trend = 0.49	Pooled analysis of Asian prospective cohort studies showed that poultry consumption was not associated with CVD mortality	Serious
Rohrmann et al. 2013	**Median intakes:** Men: 15.1 g/day Women: 12.6 g/day	**Poultry and CVD mortality** **6 categories:** 0 to 4.9 g/day: 1.05 (0.96, 1.15), 5 to 9.9 g/day: 1.00 (Ref.), 10 to 19.9 g/day: 1.00 (0.92, 1.09), 20 to 39.9 g/day: 0.92 (0.83, 1.01), 40 to 79.9 g/day: 0.90 (0.81, 1.01), 80+ g/day: 0.94 (0.73, 1.21) **Per 50 g/day:** Observed: 0.93 (0.85, 1.01), Calibrated: 0.84 (0.69, 1.03)	Intake of poultry was not associated with CVD mortality	Moderate
Takata et al. 2013	**Median intakes:** Women: Q1: 11.9 ± 0.15 Q5: 19.9 ± 0.15 Men: Q1: 11.9 ± 0.17 Q5: 22.3 ± 0.18	**Poultry and CVD mortality** **Women:** Q1: 1.00 (ref), Q2: 0.88 (0.75, 1.03), Q3: 1.08 (0.92, 1.28), Q4: 1.02 (0.85, 1.23), Q5: 1.03 (0.84, 1.26), *P* for trend = 0.47	There were suggestive inverse associations of poultry intake with the risk of CVD mortality among men but not among women	Moderate
		**Men:** Q1: 1.00 (ref), Q2: 0.80 (0.66, 0.97), Q3: 0.75 (0.61, 0.92), Q4: 0.67 (0.54, 0.84), Q5: 0.81 (0.65, 1.02), *P* for trend = 0.13		
van den Brandt et al. 2019	**Median intakes:** (g/day): 0: 0 <10: 4.3 <20: 13.2 20+: 22.8	**Poultry and CVD mortality,** **4 categories:** 0 g/day: 1 (ref), <10 g/day: 0.94 (0.78–1.12), 10 to 20 g/day: 0.87 (0.73–1.05), >20 g/day: 0.89 (0.75–1.06), *P* trend = 0.183 **Per 50 g/day:** 0.97 (0.77–1.22)	There were no associations of poultry intake with the risk of CVD mortality	Serious
**T2D incidence**				
Du et al. 2020	Servings per day. Consumption weekly, monthly, and never/rarely	HR 0.96 [95% CI: 0.83, 1.12] per 50 g/day intake	There was no significant association between diabetes and poultry intake	Moderate
InterAct. 2013	**Mean intakes (SEM):** Q1: 8.7 (0.4) g/day Q2: 15.7 (0.4) g/day Q3: 20.6 (0.4) g/day Q4: 26.1 (0.4) g/day Q5: 37.7 (0.4) g/day	**Women:** HR 1.20; 95% CI: 1.07, 1.34 per 50 g/day intake **Men:** HR 0.94; 95% CI: 0.85, 1.03 per 50 g/day intake **Total:** HR 1.03; 95% CI: 0.95, 1.11 per 50 g/day intake	In women, poultry intake was associated with higher TD2 risk	Moderate
Kurotani et al. 2013	**Median intakes:** **Men:** Q1: 0.0 g Q2: 5.1 g Q3: 9.6 g Q4: 20.1 g	**Men:** Q1 versus Q4: 1.03 (95% CI: 0.81, 1.30) **Women:** Q1 versus Q4: 0.97 (95% CI: 0.74, 1.27)	Intakes of poultry were not associated with the risk of diabetes in either men or women	Serious
	**Women:** Q1: 0.0 g Q2: 4.5 g Q3: 8.6 g Q4: 17.8 g			
Männistö et al. 2010	**Median intakes:** Q1: 2 g/day Q2: 8 g/day Q3: 10 g/day Q4: 14 g/day Q5: 14 g/day	**Q1 versus Q5:** 1.01 (95% CI: 0.85, 1.21)	No association was found between poultry and the risk of type 2 diabetes	Serious
Montonen et al. 2005	**Mean intakes (SD):** Non-cases: 2.6 (9.3) g/day Cases: 2.6 (13.1) g/day	**Q4 versus Q1:** 0.71 (95% CI: 0.54–0.94; *P* < 0.01)	Poultry intake was inversely associated with risk of type II diabetes	Serious
Steinbrecher et al. 2011	**Median intakes** (g/4,184 kJ/day): **Men:** **Fresh poultry:** Q1: 5.98 Q2: 11.65 Q3: 16.83 Q4: 23.60 Q5: 38.18 **Processed poultry:** Q1: 0.00 Q2: 0.11 Q3: 0.53 Q4: 1.20 Q5: 2.85	**Men:** Processed poultry: 1.30 (95% CI: 1.17, 1.44), unprocessed poultry: 1.06 (95% CI: 0.96, 1.18) **Women:** Processed poultry: 1.23 (95% CI: 1.10, 1.38), unprocessed poultry: 1.01 (95% CI: 0.90, 1.14)	Processed poultry was associated with an increased risk of diabetes. Fresh poultry consumption was not associated with diabetes risk	Serious
	**Women:** **Fresh poultry:** Q1: 6.46 Q2: 12.65 Q3: 18.37 Q4: 26.40 Q5: 43.24 **Processed poultry:** Q1: 0.00 Q2: 0.10 Q3: 0.42 Q4: 1.06 Q5: 2.42			
Talaei et al. 2017	**Mean intake (SD):** Q1: 4.1 (6.2) Q4: 40.9 (15.5)	Q4 versus Q1: 1.15 (95% CI: 1.06, 1.24)	Poultry intake was associated with a higher risk of T2D	Moderate
van Woudenbergh et al. 2012	**Median intakes:** 0: 0 g/day >0–≤9.1: 6.3 g/day >9.1–≤18.0: 13.9 g/day >18.0: 27.6 g/day	Highest cat versus lowest cat: 0.95 [0.74–1.22]	Intake of poultry was not associated with the risk of type 2 diabetes	Serious
Villegas et al. 2006	No information given	**Unprocessed poultry:** Q5 versus Q1: 0.79 (95% CI: 0.67–0.92)	Consumption of unprocessed poultry was associated with lower risk of type 2 diabetes	Moderate

### Prospective cohort studies

Three prospective cohort studies investigated the associations between white meat intake and coronary heart disease (**CHD) incidence**. While the study by Haring et al. (36) indicated a lower risk, the studies by Bernstein et al. (35) and Key et al. (37) showed no statistically significant associations (Table 2). One study by Park et al. (43) indicated that the intake of poultry is associated with lower risk of **incident CVD** (Table 2).

Results from Bernstein et al. (41) suggested that higher white meat intake was related to lower **stroke incidence**, which seemed to be driven by the inverse relationship observed in women. Results from Haring et al. (38) did not show such associations. Furthermore, data from Farvid et al. (39) and Sauvaget et al. (42) indicated that white meat intake was not associated with **stroke mortality** (Table 2).

**Fig. 4:**
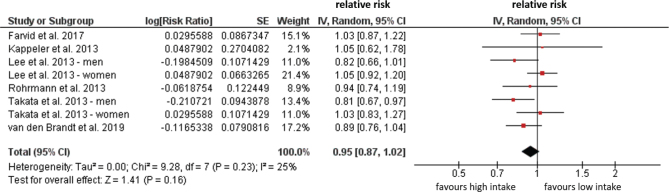
Associations between poultry intake and CVD mortality comparing highest versus lowest consumption categories.

**Fig. 5:**
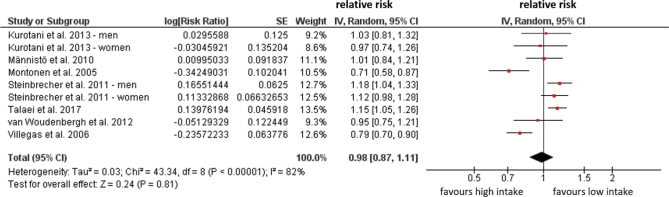
Associations between poultry intake and risk of T2D, comparing highest versus lowest consumption categories.

Two studies (Farvid et al. (39) and Nagao et al. (40)) investigated **CHD mortality** but did not find any significant associations with intake of white meat (Table 2). Six studies investigated **CVD mortality**; of which, two did a separate analysis of men and women. Takata et al. (47) found that there were suggestive inverse associations of poultry intake with risk of CVD mortality among men but not among women. Other studies (39, 44–46, 48) did not show any associations (Table 2). A meta-analysis was performed including the six above-mentioned studies for this outcome indicating no significant associations between intake of white meat and risk of CVD mortality (RR: 0.95, 95%CI: 0.87–1.02, *P* = 0.23) with low heterogeneity (*I*^2^ = 25%) (Fig. 4). Nine cohort studies investigated white meat intake and risk of **incident T2D**. In the EPIC Interact study (50), Kurotani et al. (51) and Steinbrecher et al. (54) conducted a separate analysis by sex. Steinbrecher et al. (54) further differentiated between processed and unprocessed white meat. Four studies (49, 51, 52, 56) did not show any associations between white meat intake and T2D. The EPIC InterAct study (50) indicated a higher risk in women, whereas Talaei et al. (55) showed a higher risk of T2D for all participants. However, in the study by Montonen et al., white meat intake was inversely associated with the risk of T2D (53). In the study by Steinbrecher et al. (54), processed poultry was associated with an increased risk of T2D in both men and women, whereas the intake of unprocessed poultry was not. Villegas et al. (57) reported that the consumption of unprocessed poultry was associated with lower risk of T2D (Table 2). A meta-analysis was performed excluding the studies from Du et al. (49) and EPIC Interact (50) as they did not report OR for extremes of intakes. Data on processed and unprocessed poultry from Steinrecher et al. (54) were pooled for the meta-analysis. For the remaining studies, no significant associations between high versus low intake of white meat and risk of T2D were found (RR: 0.98, 95%CI: 0.87–1.11, *P* = 0.81) with high heterogeneity (*I*^2^ = 82%) (Fig. 5).

### Certainty in the evidence

For the development of T2D, the evidence was judged as *substantial effects unlikely*. This was based on the null effects observed in one glucose metabolism RCT and a pooled RR close to 1.0 for seven meta-analyzed cohorts out of totally nine available. The main uncertainty concerning the grading was the heterogeneity observed in the meta-analysis that some included studies were classified as having serious Rob and the apparent lack of RCTs. On the other hand, it was deemed unlikely that studies in the near future would affect the conclusion.

Similarly, the evidence was judged as *substantial effects unlikely* for the outcome CVD mortality based on the pooled RR of 0.95 (95% CI 0.87–1.02) from six studies with low heterogeneity. This grading was corroborated by two cohort studies each on CHD and stroke mortality, showing null associations. The few trials identified did not support any effects of white meat on the cardiometabolic risk factors when compared to the consumption of red meat. However, all the included trials matched the dietary fat intake of the different study arms and thus do not necessarily reflect real-world conditions.

We appraised the certainty of evidence separate for the studies addressing incident diseases mainly because they were few and displayed somewhat mixed findings, and thus, the evidence was judged as *limited – no conclusion* for incident CHD, incident stroke, and incident CVD.

## Discussion

This systematic review investigated white meat consumption and risk of CVD and T2D. Taken together, based on three intervention and 23 prospective cohort studies, there was no clear indication of a role, neither beneficial nor detrimental, of increased consumption of white meat for these two disease entities.

The effects of dietary saturated fatty acids on blood lipids such as LDL and total cholesterol have been the proposed mechanism that can explain the observed associations between red meat intake and increased risk of cardiovascular disease (58, 59). As has been shown in low-fat feeding studies, the cholesterol raising effects of red meat mainly depend on its fat content and are not related so much to the protein components of red meat (60, 61). As white meat usually contains less fat than red meat, this reduction in fat intake could improve blood lipids profiles in a real world setting and therefore leads to a decreased CVD or risk of T2D (60, 61). In the present review, the effects of consumption of white meat compared to red meat on cardiovascular risk factors were investigated by three intervention studies (32–34) with a length of 4 to 5 weeks and with low to medium risk of bias. In each study, the fat content of the prescribed intervention diets of white meat and red meat was very similar, and thus, not unexpected, none of them showed any significant effects on the CVD or T2D risk factors.

The included 23 prospective cohort studies (35–57) investigated incidence and mortality of ischemic heart disease, stroke, and combined CVD as well as risk of T2D. The best evidence was available for CVD mortality (six studies) and risk of T2D (nine studies). The meta-analyses performed for these studies indicated no significant associations between intake of white meat and risk of CVD mortality (with moderately low heterogeneity) or risk for T2D (with high heterogeneity). For other cardiovascular outcomes, no meta-analyses were conducted due to a paucity of studies, and thus, the evidence was judged as limited – not conclusive.

When the results of our review are compared to recently published meta-analyses on white meat, we find good agreement. In the meta-analysis by Lupoli et al. (15), which included a total of 22 cohort studies, the consumption of white meat was related to neither lower CVD incidence nor lower CVD mortality, although it was associated with a lower total mortality, an outcome that we did not investigate in the present analysis. Another meta-analysis by Kim et al. (62) found that the relative risk related to stroke incidence and white meat to be 0.87 (95% CI: 0.78–0.97), based on two studies (38, 41), which were also included in the present review. Finally, Yang et al. included nine articles in their meta-analysis (63) and found no impact on hazard for T2D when comparing the highest to the lowest poultry intakes (HR 1.00 [95% CI: 0.93–1.07]) similar to our results.

Only few of the included studies reported the results categorized by sex, and therefore, no stratified meta-analyses were performed. Nagao et al. (40) found similar associations between white meat intake and CHD mortality in men and women, whereas Bernstein et al. (41) found lower stroke incidence in relation to white meat only in women. On the other hand, results from Lee et al. (45) and Takata et al. (47) indicated that white meat was associated with lower CVD mortality in men but not in women. Regarding risk of T2D, the EPIC Interact study reported a higher risk related to white meat intake in women only (but not in men); however, the risk estimates related to white meat were similar in men and women according to the studies from Kurotani et al. (51) and Steinbrecher et al. (54). Thus, taken together and given the possibility of sex-dependent residual confounding, the available evidence does not allow to draw a clear picture on sex differences in relation to white meat intake and disease risk.

In general, several studies on red meat intake and disease risk have reported that the risk is more related to the intake of processed meat than the unprocessed meat (19, 64, 65). In this context, it is interesting that only two of the studies (54, 57) included in the present review reported findings for unprocessed white meat, and only one differentiated between processed and unprocessed white meat. The study by Steinbrecher et al. (54) showed a higher risk for processed white meat, whereas Villegas et al. (57) showed a lower risk for unprocessed white meat.

Everything considered, the currently available evidence on white meat consumption and CVD as well as T2D does not support a protective role of white meat consumption. There were some indications of sex differences in the associations among white meat intake, stroke incidence, and CVD mortality, but of unclear relevance. Furthermore, a differentiation between unprocessed and processed white meat is necessary in future studies to shed light on potential harmful or protective effects of white meat intake.

### Strength and limitations

The strength of the current review is the extensive and elaborative methods in collecting, reviewing, and grading the currently available evidence with the aim to translate the scientific evidence into dietary recommendations relevant for public health.

The current SR did not consider substitution of red meat with white meat but only intake of white meat. This can be regarded as a limitation, as food items are usually not consumed in addition to other foods but will replace them in the diet. Replacing red meat/processed red meat with poultry has been associated with lower total mortality (66), while associations with CVD endpoints or T2D have been unclear (67–70).

A limitation for every systematic review and meta-analysis is that it is dependent on the availability and quality of relevant studies. We could not perform a meta-analysis or even a subgroup analysis for many of the intended outcomes due to the low number of studies. Furthermore, according to our assessment, 11 of 23 cohort studies had a serious risk of bias with the remaining studies having a moderate risk of bias. The number of included intervention studies was low, and although their risk of bias was judged low in two of the three studies, their study designs leveled differences in fat intake associated with red meat and white meat intake. This also decreased the likelihood of finding differences in CVD or T2D risk factors that might be observed in a real-world setting.

## Conclusion

This systematic review and meta-analysis investigated white meat consumption and risk of CVD and T2D using relevant intervention trials and prospective cohort studies. The currently available evidence does not indicate a role, either beneficial or detrimental, of white meat consumption for these diseases.
