# Supplementary material for: White meat consumption and risk of cardiovascular disease and type 2 diabetes: a systematic review and meta-analysis
Source: Food Nutr Res. 2023 Dec 28;67:10.29219/fnr.v67.9543. doi: 10.29219/fnr.v67.9543 (PMC10770644; doi:10.29219/fnr.v67.9543)
Supplement: Supplementary file 1 [file FNR-67-9543-s001.docx]

**Supplemental Table 1:** Eligibility criteria for population/participants, intervention/exposure, control, outcome, timeframe, study design and settings (PI/ECOTSS)

| **Population** | **study design** | **intervention/**  **exposure** | **comparators** | **outcomes** | **timing** | **setting** |
| --- | --- | --- | --- | --- | --- | --- |
| Healthy adults | randomized | white meat | red meat replaced | insulin resistance | ≥4 weeks | relevant for the |
|  | controlled |  | with white meat | insulin sensitivity | intervention period | general population |
|  | trials |  |  | HBA1c |  | in |
|  |  |  |  | fasting glucose |  | the Nordic and |
|  |  |  |  | fasting insulin |  | Baltic countries |
|  |  |  |  | blood lipids |  |  |
|  |  |  |  | blood pressure |  |  |
|  |  |  |  |  |  |  |
| Healthy adults | prospective | white meat | no/low consumption | major incident fatal | ≥12 months |  |
|  | cohort |  | vs. high consumption | and non-fatal | follow-up | relevant for the |
|  | studies |  |  | atherosclerotic cardiovascular |  | general population |
|  |  |  |  | diseases: |  | in |
|  |  |  |  | myocardial infarction |  | the Nordic and |
|  |  |  |  | stroke |  | Baltic countries |
|  |  |  |  | coronary heart disease |  |  |
|  |  |  |  | coronary artery bypass graft |  |  |
|  |  |  |  |  |  |  |
|  |  |  |  | CVD mortality |  |  |
|  |  |  |  |  |  |  |
|  |  |  |  | Incident T2D |  |  |

**Supplemental Table 2:** Documentation of literature research.

Documentation on the literature search for “What is the association between consumption of white meat and the incidence of or mortality from atherosclerotic cardiovascular diseases and type 2 diabetes?”

The following databases were searched:

| **Database** | **Number of retrieved references** |
| --- | --- |
| Medline (Ovid): | 2297 |
| Embase (Ovid): | 2142 |
| Cochrane Central Register of Controlled Trials (Cochrane Library, Wiley): | 340 |
| Scopus : | 3431 |
| Number of references before deduplication: | 8202 |
| Number of references after deduplication: | 5795 |

All searches were performed by Gunn Kleven, senior librarian, University of Oslo, Library of Medicine and Science on 15 October 2021

**Ovid MEDLINE(R) ALL**1946 to October 08, 2021:

Search Strategy:

| **#** | **Searches** | **Results** |
| --- | --- | --- |
| 1 | poultry/ or chickens/ or ducks/ or geese/ or turkeys/ | 156590 |
| 2 | poultry products/ | 1408 |
| 3 | (poultry or poultries or chicken* or (white adj2 meat) or ((goose* or geese* or duck* or turkey*) adj3 meat)).ti,ab,kf. | 126175 |
| 4 | or/1-3 | 205793 |
| 5 | Cardiovascular Diseases/ or Atherosclerosis/ or exp Myocardial Infarction/ or exp Stroke/ or exp Coronary Disease/ or exp Coronary Artery Bypass/ | 723024 |
| 6 | ((ACA or anterior cerebral artery or anterior cerebral circulation or anterior choroidal artery or brain or brain stem or brainstem or brain venous or cerebral or heubner* artery or MCA or middle cerebral artery or myocardial or PCA or posterior cerebral artery or posterior choroidal artery or subcortical) adj2 infarct*).ti,ab,kf. | 239800 |
| 7 | ((anterior cerebral artery or basilar or benedict or claude or coronary-subclavian steal or dorsolateral medullary or foville or lateral bulbar or lateral medullary or middle cerebral artery or millard-gublar or posterior cerebral artery or posterior inferior cerebellar artery or wallenberg* or weber) adj2 syndrome*).ti,ab,kf. | 3550 |
| 8 | ((brain vascular or cerebrovascular) adj2 accident*).ti,ab,kf. | 7734 |
| 9 | ((coronary artery or aortocoronary) adj2 bypass*).ti,ab,kf. | 45994 |
| 10 | (coronary adj3 (aneurysm* or arterioscleros#s or artery anastomos#s or disease* or occlusion* or restenos#s or stenos#s or syndrome* or thrombos#s or vasospasm*)).ti,ab,kf. | 223184 |
| 11 | (apoplex* or atherogenesis or atheroscleros#s or cardiogenic shock or heart attack* or middle cerebral artery thrombosis or stroke*).ti,ab,kf. | 418774 |
| 12 | ((cardio* or cerebral* or cerebro* or coronary or CVD or heart* or myocardial) adj3 (death? or mortalit*)).ti,ab,kf. | 94622 |
| 13 | or/5-12 | 1090491 |
| 14 | exp Diabetes Mellitus, Type 2/ or exp Insulin Resistance/ or C-Peptide/ or Glucose Intolerance/ or Glycated Hemoglobin A/ or Blood Glucose/ or Hyperglycemia/ or Blood Pressure/ or Hypertension/ or Lipids/ or exp Triglycerides/ or exp Apolipoproteins/ or Cholesterol/ or Cholesterol, HDL/ or Cholesterol, LDL/ or Cholesterol, VLDL/ | 1055128 |
| 15 | (diabet* adj3 ("2" or "type II" or Adult-Onset or Non Insulin or NonInsulin)).ti,ab,kf. | 181969 |
| 16 | (DM2 or NIDDM or IIDM or MODY or T2DM).ti,ab,kf. | 35810 |
| 17 | (blood adj2 (glucose or sugar*)).ti,ab,kf. | 94354 |
| 18 | (lipid* adj2 (blood or level or profile*)).ti,ab,kf. | 56650 |
| 19 | (blood pressure or cardiometabolic syndrome* or C-peptide or cholesterol or HDL or LDL or VLDL or connecting peptide or diastolic pressure or dysmetabolic syndrome* or glucose intolerance* or HOMA-IR or hyperglycemia* or hypertension or insulin resistance or insulin sensitivity or metabolic syndrome* or metabolic cardiovascular syndrome* or pulse pressure or reaven syndrome X or systolic pressure).ti,ab,kf. | 1045756 |
| 20 | ((glycated or glycosylated) adj2 (haemoglobin* or hemoglobin*)).ti,ab,kf. | 24621 |
| 21 | (glycohemoglobin A or Hb A1 or HbA1 or Hb A1a-1 or Hb A1a-2 or Hb A1a+b or Hb A1b or Hb A1c or HbA1c or "hemoglobin A(1)" or hemoglobin A1C).ti,ab,kf. | 47881 |
| 22 | (Apo-B or ApoA or ApoA-II or Apo A-V or Apo A1 or Apo A2 or Apo A5 or APOA5 or Apo AI or ApoB or ApoB48 or ApoC or Apo C or ApoC2 or Apo D or ApoD or ApoE or Apo E or APOE-epsilon* or ApoE2 or Apo E2 or Apo E3 or ApoE3 or Apo E4 or ApoE4 or ApoL or ApoL1 or apolipoprotein* or apoprotein* or enzactin or glycerol trioleate or proapolipoprotein* or triacetin or triacetyl-glycerol* or triacetylglycerol* or triacylglycerol* or trielaidin or triglyceride* or trioleate-glycerin or triolein or trioleoylglycerol or trioleyl glycerol*).ti,ab,kf. | 195515 |
| 23 | or/14-22 | 1645132 |
| 24 | or/13,23 | 2454441 |
| 25 | and/4,24 | 8830 |
| 26 | 25 not (exp "Animals"/ not (exp "Animals"/ and "Humans"/)) | 2387 |
| 27 | 26 not (editorial or interview or letter or comment or congress or legal case or meeting abstract).pt. | 2297 |

**Embase Classic+Embase**1947 to 2021 October 08

Search Strategy:

| **#** | **Searches** | **Results** |
| --- | --- | --- |
| 1 | white meat/ or poultry meat/ or chicken meat/ or turkey meat/ | 2585 |
| 2 | poultry/ or duck/ or goose/ | 33509 |
| 3 | (poultry or poultries or chicken* or (white adj2 meat*) or ((goose* or geese* or duck* or turkey*) adj3 meat*)).ti,ab,kw. | 143481 |
| 4 | or/1-3 | 160837 |
| 5 | cardiovascular disease/ or atherosclerosis/ or exp heart infarction/ or exp cerebrovascular accident/ or exp coronary artery disease/ or coronary artery bypass graft/ | 1277907 |
| 6 | ((ACA or anterior cerebral artery or anterior cerebral circulation or anterior choroidal artery or brain or brain stem or brainstem or brain venous or cerebral or heubner* artery or MCA or middle cerebral artery or myocardial or PCA or posterior cerebral artery or posterior choroidal artery or subcortical) adj2 infarct*).ti,ab,kw. | 351512 |
| 7 | ((anterior cerebral artery or basilar or benedict or claude or coronary-subclavian steal or dorsolateral medullary or foville or lateral bulbar or lateral medullary or middle cerebral artery or millard-gublar or posterior cerebral artery or posterior inferior cerebellar artery or wallenberg* or weber) adj2 syndrome*).ti,ab,kw. | 4884 |
| 8 | ((brain vascular or cerebrovascular) adj2 accident*).ti,ab,kw. | 12236 |
| 9 | ((coronary artery or aortocoronary) adj2 bypass*).ti,ab,kw. | 60220 |
| 10 | (coronary adj3 (aneurysm* or arterioscleros#s or artery anastomos#s or disease* or occlusion* or restenos#s or stenos#s or syndrome* or thrombos#s or vasospasm*)).ti,ab,kw. | 328616 |
| 11 | (apoplex* or atherogenesis or atheroscleros#s or cardiogenic shock or heart attack* or middle cerebral artery thrombosis or stroke*).ti,ab,kw. | 662360 |
| 12 | ((cardio* or cerebral* or cerebro* or coronary or CVD or heart* or myocardial) adj3 (death? or mortalit*)).ti,ab,kw. | 154622 |
| 13 | or/5-12 | 1741687 |
| 14 | non insulin dependent diabetes mellitus/ or insulin resistance/ or c peptide/ or glucose intolerance/ or hemoglobin a1c/ or glucose blood level/ or hyperglycemia/ or blood pressure/ or hypertension/ or lipid blood level/ or exp apolipoprotein/ or exp high density lipoprotein cholesterol/ or low density lipoprotein cholesterol/ or very low density lipoprotein cholesterol/ | 1672040 |
| 15 | (diabet* adj3 ("2" or "type II" or Adult-Onset or Non Insulin or NonInsulin)).ti,ab,kw. | 275932 |
| 16 | (DM2 or NIDDM or IIDM or MODY or T2DM).ti,ab,kw. | 58691 |
| 17 | (blood adj2 (glucose or sugar*)).ti,ab,kw. | 149406 |
| 18 | (lipid* adj2 (blood or level or profile*)).ti,ab,kw. | 80993 |
| 19 | (blood pressure or cardiometabolic syndrome* or C-peptide or cholesterol or HDL or LDL or VLDL or connecting peptide or diastolic pressure or dysmetabolic syndrome* or glucose intolerance* or HOMA-IR or hyperglycemia* or hypertension or insulin resistance or insulin sensitivity or metabolic syndrome* or metabolic cardiovascular syndrome* or pulse pressure or reaven syndrome X or systolic pressure).ti,ab,kw. | 1568592 |
| 20 | ((glycated or glycosylated) adj2 (haemoglobin* or hemoglobin*)).ti,ab,kw. | 32386 |
| 21 | (glycohemoglobin A or Hb A1 or HbA1 or Hb A1a-1 or Hb A1a-2 or Hb A1a+b or Hb A1b or Hb A1c or HbA1c or "hemoglobin A(1)" or hemoglobin A1C).ti,ab,kw. | 95737 |
| 22 | (Apo-B or ApoA or ApoA-II or Apo A-V or Apo A1 or Apo A2 or Apo A5 or APOA5 or Apo AI or ApoB or ApoB48 or ApoC or Apo C or ApoC2 or Apo D or ApoD or ApoE or Apo E or APOE-epsilon* or ApoE2 or Apo E2 or Apo E3 or ApoE3 or Apo E4 or ApoE4 or ApoL or ApoL1 or apolipoprotein* or apoprotein* or enzactin or glycerol trioleate or proapolipoprotein* or triacetin or triacetyl-glycerol* or triacetylglycerol* or triacylglycerol* or trielaidin or triglyceride* or trioleate-glycerin or triolein or trioleoylglycerol or trioleyl glycerol*).ti,ab,kw. | 275081 |
| 23 | or/14-22 | 2449070 |
| 24 | or/13,23 | 3668011 |
| 25 | and/4,24 | 7300 |
| 26 | (exp animal/ or exp animal model/ or nonhuman/) not exp human/ | 7463613 |
| 27 | 25 not 26 | 2878 |
| 28 | 27 not (Conference abstract or Conference paper or Conference review or Editorial or Letter or Note or Short survey).pt. | 2134 |

**Cochrane Central Register of Controlled Trials:**

| #1 | [mh "poultry"] OR [mh "chickens"] OR [mh "ducks"] OR [mh "geese"] OR [mh "turkeys"] OR [mh "poultry products"] | 202 |
| --- | --- | --- |
| #2 | (poultry or poultries or (white NEAR/2 meat*) or chicken*):ti,ab,kw | 1730 |
| #3 | ((goose* or geese* or duck* or turkey*) NEAR/3 meat*):ti,ab,kw | 34 |
| #4 | #1 OR #2 OR #3 | 1778 |
| #5 | [mh ^"Cardiovascular Diseases"] OR [mh ^Atherosclerosis] OR [mh "Myocardial Infarction"] OR [mh Stroke] OR [mh "Coronary Disease"] OR [mh "Coronary Artery Bypass"] | 45373 |
| #6 | ((ACA OR "anterior cerebral artery" OR "anterior cerebral circulation" OR "anterior choroidal artery" OR brain OR "brain stem" OR brainstem OR "brain venous" OR cerebral OR heart OR (heubner* NEXT artery) OR MCA OR "middle cerebral artery" OR myocardial OR PCA OR "posterior cerebral artery" OR "posterior choroidal artery" OR subcortical) NEAR/2 infarct*):ti,ab,kw | 40735 |
| #7 | (("anterior cerebral artery" OR basilar OR benedict OR claude OR "coronary-subclavian steal" OR "dorsolateral medullary" OR foville OR "lateral bulbar" OR "lateral medullary" OR "middle cerebral artery" OR millard-gublar OR "posterior cerebral artery" OR "posterior inferior cerebellar artery" OR wallenberg* OR weber) NEAR/2 syndrome*):ti,ab,kw | 48 |
| #8 | (("brain vascular" OR cerebrovascular) NEAR/2 accident*):ti,ab,kw | 14172 |
| #9 | (("coronary artery" OR aortocoronary) NEAR/2 bypass*):ti,ab,kw | 12442 |
| #10 | (coronary NEAR/3 (aneurysm* OR arterioscleros?s OR "artery anastomos")):ti,ab,kw | 195 |
| #11 | (apoplex* OR atherogenesis OR atheroscleros?s OR "cardiogenic shock" OR ("heart" NEXT attack*) OR "middle cerebral artery thrombosis" OR stroke*):ti,ab,kw | 71073 |
| #12 | ((cardio* OR cerebral* OR cerebro* OR coronary OR CVD OR heart* OR myocardial) NEAR/3 (death? OR mortalit*)):ti,ab,kw | 23843 |
| #13 | [mh "Diabetes Mellitus, Type 2"] OR [mh "Insulin Resistance"] OR [mh ^"C-Peptide"] OR [mh ^"Glucose Intolerance"] OR [mh ^"Glycated Hemoglobin A"] OR [mh ^"Blood Glucose"] OR [mh ^Hyperglycemia] OR [mh ^"Blood Pressure"] OR [mh ^Hypertension] OR [mh ^Lipids] OR [mh Triglycerides] OR [mh Apolipoproteins] OR [mh ^Cholesterol] OR [mh ^"Cholesterol, HDL"] OR [mh ^"Cholesterol, LDL"] OR [mh ^"Cholesterol, VLDL"] | 79052 |
| #14 | (diabet* NEAR/3 (2 OR "type II" OR "Adult-Onset" OR "Non Insulin" OR NonInsulin)):ti,ab,kw | 47268 |
| #15 | (DM2 OR NIDDM OR IIDM OR MODY OR T2DM):ti,ab,kw | 8351 |
| #16 | (blood NEAR/2 (glucose OR sugar*)):ti,ab,kw | 43040 |
| #17 | (lipid* NEAR/2 (blood OR level OR profile*)):ti,ab,kw | 20356 |
| #18 | ("blood pressure" OR (cardiometabolic NEXT syndrome*) OR "C-peptide" OR cholesterol OR HDL OR LDL OR VLDL OR "connecting peptide" OR "diastolic pressure" OR (dysmetabolic NEXT syndrome*) OR (glucose NEXT intolerance*) OR "HOMA-IR" OR hyperglycemia* OR hypertension OR "insulin resistance" OR "insulin sensitivity" OR (metabolic NEXT syndrome*) OR ("metabolic cardiovascular" NEXT syndrome*) OR "pulse pressure" OR "reaven syndrome X" OR "systolic pressure"):ti,ab,kw | 182397 |
| #19 | ((glycated or glycosylated) NEAR/2 (haemoglobin* or hemoglobin*)):ti,ab,kw | 11538 |
| #20 | ("glycohemoglobin A" OR "Hb A1" OR HbA1 OR "Hb A1a-1" OR "Hb A1a-2" OR "Hb A1a+b" OR "Hb A1b" OR "Hb A1c" OR HbA1c OR "hemoglobin A(1)" OR "hemoglobin A1C"):ti,ab,kw | 23232 |
| #21 | ("Apo-B" OR ApoA OR "ApoA-II" OR "Apo A-V" OR "Apo A1" OR "Apo A2" OR "Apo A5" OR APOA5 OR "Apo AI" OR ApoB OR ApoB48 OR ApoC OR "Apo C" OR ApoC2 OR "Apo D" OR ApoD OR ApoE OR "Apo E" OR (APOE NEXT epsilon*) OR ApoE2 OR "Apo E2" OR "Apo E3" OR ApoE3 OR "Apo E4" OR ApoE4 OR ApoL OR ApoL1 OR apolipoprotein* OR apoprotein* OR enzactin OR "glycerol trioleate" OR proapolipoprotein* OR triacetin OR (triacetyl NEXT glycerol*) OR triacetylglycerol* OR triacylglycerol* OR trielaidin OR triglyceride* OR (trioleate NEXT glycerin) OR triolein OR trioleoylglycerol OR ("trioleyl" NEXT glycerol*)):ti,ab,kw | 30119 |
| #22 | #5 OR #6 OR #7 OR #8 OR #9 OR #10 OR #11 OR #12 OR #13 OR #14 OR #15 OR #16 OR #17 OR #18 OR #19 OR #20 OR #21 | 324211 |
| #23 | #4 AND #22 | 343 |

**Scopus:**

((TITLE-ABS-KEY(poultry OR poultries OR (white W/2 meat*) OR ((goose* OR geese* OR duck* OR turkey*) W/2 meat*))) OR (TITLE-ABS-KEY(chicken* W/2 (meat* OR breast*)))) AND ((TITLE-ABS-KEY((ACA or "anterior cerebral artery" or "anterior cerebral circulation" or "anterior choroidal artery" or brain or "brain stem" or brainstem or "brain venous" or cerebral or heart or "heubner* artery" or MCA or "middle cerebral artery" or myocardial or PCA or "posterior cerebral artery" or "posterior choroidal artery" or subcortical) W/1 infarct*)) OR (TITLE-ABS-KEY(("anterior cerebral artery" or basilar or benedict or claude or "coronary-subclavian steal" or "coronary subclavian steal" or "dorsolateral medullary" or foville or "lateral bulbar" or "lateral medullary" or "middle cerebral artery" or "millard-gublar" or "millard gublar" or "posterior cerebral artery" or "posterior inferior cerebellar artery" or wallenberg* or weber) W/1 syndrome*)) OR (TITLE-ABS-KEY(("brain vascular" or cerebrovascular) W/1 accident*)) OR (TITLE-ABS-KEY(("coronary artery" or aortocoronary) W/1 bypass*)) OR (TITLE-ABS-KEY(coronary W/2 (aneurysm* or arteriosclerosis or "artery anastomosis" or disease* or occlusion* or restenosis or stenosis or syndrome* or thrombosis or vasospasm*))) OR (TITLE-ABS-KEY((apoplex* or atherogenesis or atherosclerosis or "cardiogenic shock" or "heart attack*" or "middle cerebral artery thrombosis" or stroke*))) OR (TITLE-ABS-KEY((cardio* or cerebral* or cerebro* or coronary or CVD or heart* or myocardial) W/2 (death* or mortalit*))) OR (TITLE-ABS-KEY(diabet* W/2 ("2" or "type II" or "Adult-Onset" or "Adult Onset" or "Non Insulin" or NonInsulin))) OR (TITLE-ABS-KEY(DM2 or NIDDM or IIDM or MODY or T2DM)) OR (TITLE-ABS-KEY(blood W/1 (glucose or sugar*))) OR (TITLE-ABS-KEY(lipid* W/1 (blood or level or profile*))) OR (TITLE-ABS-KEY(("blood pressure" or "cardiometabolic syndrome*" or "C-peptide" or "C peptide" or cholesterol or HDL or LDL or VLDL or "connecting peptide" or "diastolic pressure" or "dysmetabolic syndrome*" or "glucose intolerance*" or "HOMA-IR" or "HOMA IR" or hyperglycemia* or hypertension or "insulin resistance" or "insulin sensitivity" or "metabolic syndrome*" or "metabolic cardiovascular syndrome*" or "pulse pressure" or "reaven syndrome X" or "systolic pressure"))) OR (TITLE-ABS-KEY((glycated or glycosylated) W/1 (haemoglobin* or hemoglobin*))) OR (TITLE-ABS-KEY(("glycohemoglobin A" or "Hb A1" or HbA1 or "Hb A1a-1" or "Hb A1a-2" or "Hb A1a b" or "Hb A1b" or "Hb A1c" or HbA1c or "hemoglobin A(1)" or "hemoglobin A1C"))) OR (TITLE-ABS-KEY(("Apo-B" or "Apo B" or ApoA or "ApoA-II" or "ApoA II" or "Apo A-V" or "Apo A V" or "Apo A1" or "Apo A2" or "Apo A5" or APOA5 or "Apo AI" or ApoB or ApoB48 or ApoC or "Apo C" or ApoC2 or "Apo D" or ApoD or ApoE or "Apo E" or "APOE-epsilon*" or "APOE epsilon*" or ApoE2 or "Apo E2" or "Apo E3" or ApoE3 or "Apo E4" or ApoE4 or ApoL or ApoL1 or apolipoprotein* or apoprotein* or enzactin or "glycerol trioleate" or proapolipoprotein* or triacetin or "triacetyl-glycerol*" or "triacetyl glycerol*" or triacetylglycerol* or triacylglycerol* or trielaidin or triglyceride* or "trioleate-glycerin" or "trioleate glycerin" or triolein or trioleoylglycerol or "trioleyl glycerol*")))) AND ( EXCLUDE ( DOCTYPE,"re" ) OR EXCLUDE ( DOCTYPE,"cp" ) OR EXCLUDE ( DOCTYPE,"ch" ) OR EXCLUDE ( DOCTYPE,"le" ) OR EXCLUDE ( DOCTYPE,"no" ) OR EXCLUDE ( DOCTYPE,"ed" ) OR EXCLUDE ( DOCTYPE,"sh" ) OR EXCLUDE ( DOCTYPE,"cr" ) OR EXCLUDE ( DOCTYPE,"bk" ) )

**Supplemental Table 3** World Cancer Research Fund’s grading23.

| **Grading of evidence categorized into five possible classes** |
| --- |
| A convincing body of evidence was established as strong enough to support a causal relationship or lack of a relationship in which several conditions are met, including evidence coming from more than one study type. |
| A probable body of evidence was supported when strong enough to support a probable causal relationship and there was evidence from at least two independent cohort studies, no unexplained heterogeneity between or within study types, good-quality studies to confidentially exclude possible random or systematic errors, and evidence for biological plausibility. |
| A limited – suggestive body of evidence was supported when there was evidence from at least two independent cohort studies, a consistent direction of effect, and evidence for biological plausibility. |
| A limited – no conclusion evidence was established if the evidence is so limited that no firm conclusion could be made. |
| Evidence strong enough to support a convincing absence of a causal relationship was considered substantial effects unlikely. |
